# METTL3 restricts RIPK1-dependent cell death via the ATF3-cFLIP axis in the intestinal epithelium

**DOI:** 10.1186/s13619-024-00197-8

**Published:** 2024-08-02

**Authors:** Meimei Huang, Xiaodan Wang, Mengxian Zhang, Yuan Liu, Ye-Guang Chen

**Affiliations:** 1 The State Key Laboratory of Membrane Biology, Tsinghua-Peking Center for Life Sciences, School of Life Sciences, Tsinghua University, Beijing, 100084 China; 2 The MOE Basic Research and Innovation Center for the Targeted Therapeutics of Solid Tumors, School of Basic Medical Sciences, Jiangxi Medical College, Nanchang University, Nanchang, 330031 China; 3 Guangzhou National Laboratory, Guangzhou, 510700 China

**Keywords:** Apoptosis, Necroptosis, Intestinal homeostasis, *Mettl3*, m^6^A modification, RIPK1

## Abstract

Intestinal epithelial cells (IECs) are pivotal for maintaining intestinal homeostasis through self-renewal, proliferation, differentiation, and regulated cell death. While apoptosis and necroptosis are recognized as distinct pathways, their intricate interplay remains elusive. In this study, we report that *Mettl3*-mediated m^6^A modification maintains intestinal homeostasis by impeding epithelial cell death. *Mettl3* knockout induces both apoptosis and necroptosis in IECs. Targeting different modes of cell death with specific inhibitors unveils that RIPK1 kinase activity is critical for the cell death triggered by *Mettl3* knockout. Mechanistically, this occurs via the m^6^A-mediated transcriptional regulation of *Atf3*, a transcription factor that directly binds to *Cflar*, the gene encoding the anti-cell death protein cFLIP. cFLIP inhibits RIPK1 activity, thereby suppressing downstream apoptotic and necroptotic signaling. Together, these findings delineate the essential role of the METTL3-ATF3-cFLIP axis in homeostatic regulation of the intestinal epithelium by blocking RIPK1 activity.

**Supplementary Information:**

The online version contains supplementary material available at 10.1186/s13619-024-00197-8.

## Background

The small intestine epithelium comprises a monolayer of cells organized into distinct structures known as crypts and villi. At the crypt, intestinal stem cells (ISCs) undergo rapid renewal and differentiation, replenishing the stem cell pool and giving rise to differentiated cells (Barker 2014). Homeostasis in the intestinal epithelium relies on continuous renewal and regulated cell death (Okumura and Takeda 2017; Wang et al. 2021). Aberrant increase in cell death is a major contributor to structural disruption in the epithelium, leading to pathological conditions such as inflammatory bowel diseases (IBD) and infectious colitis (Chan 2012; Wang et al. 2022).

It has been reported that intestinal epithelial cells can undergo different types of cell death, including anoikis, apoptosis, necroptosis, ferroptosis and pyroptosis (Patankar and Becker 2020; Subramanian et al. 2020). Receptor-interacting protein kinase (RIPK) 1 is a pivotal protein kinase that mediates both apoptosis and necroptosis (Christofferson et al. 2014; Xu et al. 2021). Upon activation, RIPK1 interacts with FADD, TRADD, and caspase 8 to form the complex II a, promoting RIPK1-dependent apoptosis (Micheau and Tschopp 2003). In the presence of caspase inhibitors, activated RIPK1 binds to RIPK3 to form the complex II b, leading to the activation of Mixed-lineage kinase domain-like protein (MLKL) and subsequent necroptosis (Yuan et al. 2019). In the small intestinal epithelium, inhibition of RIPK1 kinase activity suppresses intestinal epithelial cell death and prevents Paneth cell loss in the mice with combined RelA, c-Rel, and RelB deficiency in IECs or IEC-specific knockout (KO) of NEMO (Vlantis et al. 2016). Conversely, specific deficiency of *Ripk1* in IECs leads to extensive apoptosis and necroptosis through its scaffold function but not kinase function (Dannappel et al. 2014; Takahashi et al. 2014). Although post-translational modification regulation of RIPK1 has been elucidated in the small intestinal epithelium (Chan 2014), the role of mRNA modifications in intestinal cell death is poorly understood.

N6-adenomethylation (m^6^A) is the most prevalent post-transcriptional modification of eukaryotic RNAs, constituting 0.4%~0.6% of all adenosine nucleotides in mammalian RNAs (Frye et al. 2018). m^6^A methylation is facilitated by a multi-subunit m^6^A methyltransferase complex, composed of methyltransferase-like 3 (METTL3) and 14 (METTL14) (Liu et al. 2014). Removal of m^6^A methylation is orchestrated by RNA demethylases, including FTO (Fat mass and obesity-associated protein) and ALKBH5 (AlkB homolog 5) (Jia et al. 2011; Zheng et al. 2013). Various “reader” proteins mediate the execution of m^6^A functionality, such as RNA stability, splicing, nuclear export, or translation (Zaccara et al. 2019; Zhao et al. 2017). Emerging evidence underscores the indispensability of m^6^A modification in embryonic development, organ growth, and stem cell maintenance (Geula et al. 2015; Li et al. 2018; Liu et al. 2023). m^6^A function in intestine is also appreciated, including ISCs maintenance, epithelial homeostasis and regeneration (Du et al. 2022; Han et al. 2020; Liu et al. 2023; Zhang et al. 2022). However, the role of *Mettl3*-mediated m^6^A in regulating cell death of the small intestinal epithelium remains elusive.

Here, we report that *Mettl3*-mediated m^6^A modification is involved in both apoptosis and necroptosis in the small intestinal epithelium. Using mouse and organoid models, we demonstrate that the METTL3-ATF3-cFLIP-RIPK1 axis balances cell survival and death. Mechanistically, ATF3, acting downstream of *Mettl3*, stimulates the expression of the anti-cell death protein cFLIP, leading to inhibition of RIPK1 and thus cell survival.

## Results

### Mettl3 deletion induces death of intestinal epithelial cells

In our previous study, we unveiled that METTL3 plays a crucial role in maintaining ISCs in the intestinal epithelium. IEC-specific deletion of *Mettl3* compromises stemness without affecting proliferation (Liu et al. 2023). Among the *Mettl3*-mediated m^6^A modified mRNAs, four encoded transcriptional factors that regulated stemness gene expression in ISCs. Their ectopic expression restored stemness gene expression but could not rescue cell death (Liu et al. 2023). These findings suggest that, beyond its role in stemness regulation, *Mettl3*-mediated m^6^A methylation is also critical in cell death regulation. Indeed, gene ontology (GO) analysis revealed the genes involved in programmed cell death pathways were enriched among the up-regulated genes in *Mettl3*-KO IECs (Fig. S1A).

**Fig. 1:**
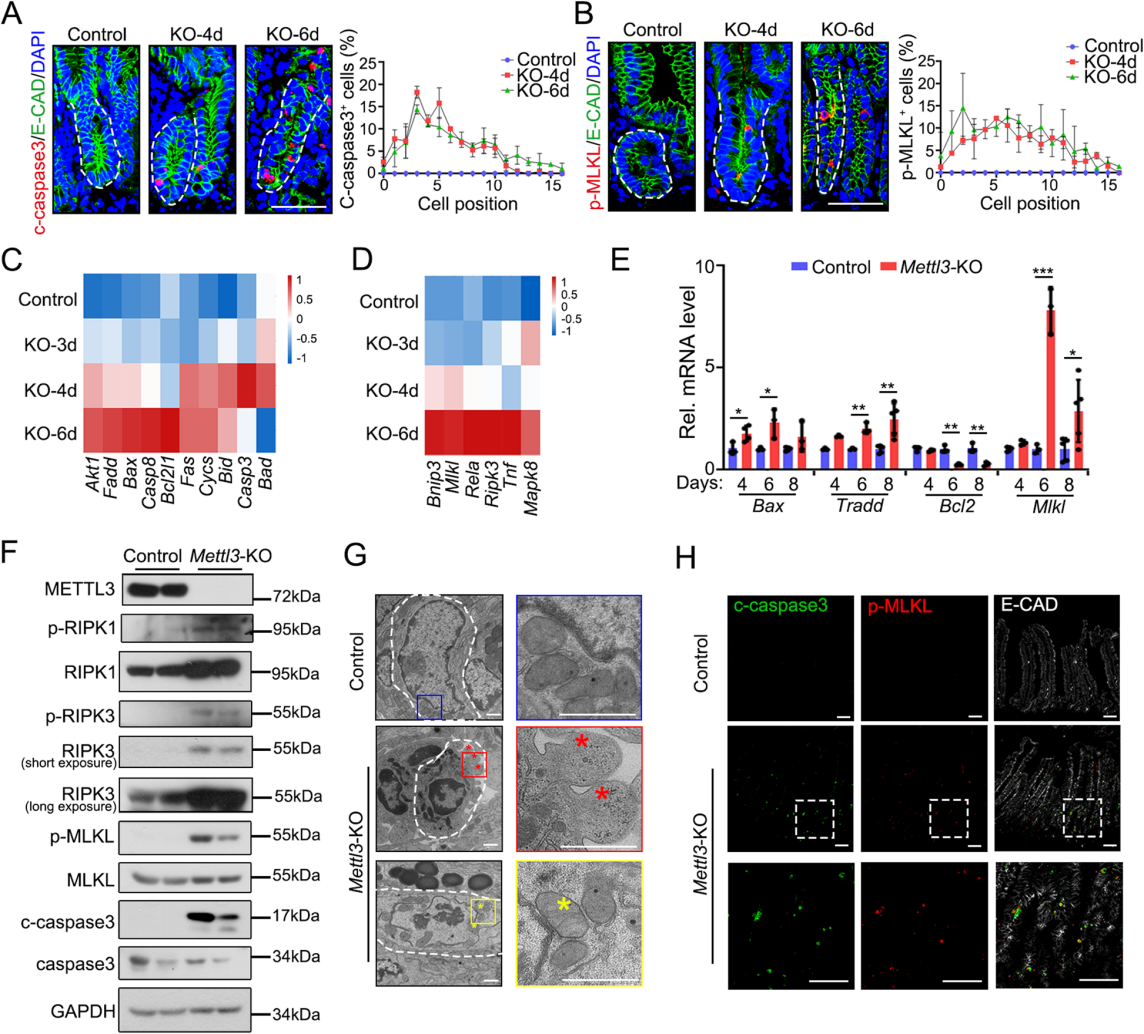
*Mettl3* deletion results in cell death in the intestinal epithelium. **A**, **B** Representative images (left) and quantification (right) of cleaved caspase3^+^ cell (**A**) and p-MLKL^+^ cell (**B**) position in jejunum crypts of control (*Mettl3*^*fl/fl*^) and *Mettl3*-KO (*Vil-CreERT2*;*Mettl3*^*fl/fl*^) mice at the indicated time after TAM injection. The cell boundary was defined by E-cadherin. *n* = 3 mice/group. **C**, **D** Expression heatmap of apoptosis (**C**) and necroptosis (**D**) -associated genes in Lgr5^+^ ISCs of control and *Mettl3*-KO mice at day 3, 4, 6 post tamoxifen (dpt), based on bulk RNA-seq. **E** qPCR shows the expression of apoptosis and necroptosis-associated genes in the organoids derived from *Vil-CreERT2*;*Mettl3*^*fl/fl*^ mice at the indicated time after treated with EtOH or 4-OHT for 2 days. Data were from three independent experiments. **F** Immunoblotting analyses of apoptosis and necroptosis-associated effector proteins in crypt epithelium isolated from control and *Mettl3*-KO mice at 6 dpt. GAPDH, loading control. Each lane represents one mice sample. *n* = 2 mice/group. **G** Representative electron microscopy images of cell death in jejunum crypts in *Mettl3*-KO mice at 4-dpt. Red asterisk marks apoptotic bodies of apoptotic cells. Yellow asterisk marks swelling mitochondrion, indicating necroptosis. *n* = 2 mice/group. **H** Immunofluorescence co-staining of cleaved caspase3 and p-MLKL in the jejunum of control and *Mettl3*-KO mice at 6dpt. *n* = 3 mice/group. All the data represent mean ± SD. The data were analyzed by Two-way ANOVA (**E**). *P < 0.05, **P < 0.01 and ***P < 0.001. Scale bars: 50 μm (**A**, **B**, **H**), 1 μm (**G**). Nuclei were counter-stained with DAPI

Various programmed cell death mechanisms have been identified in IECs. In addition to apoptosis, necroptosis, pyroptosis, and ferroptosis have been reported in the intestinal epithelium (Patankar and Becker 2020). We first examined the mode of cell death occurring in the *Mettl3*-KO intestinal epithelium. The hallmarks of apoptosis and necroptosis, cleaved caspase3 (c-caspase 3) and phosphorylated MLKL (p-MLKL), were observed in *Mettl3*-KO intestinal epithelium at day 4 and day 6 (Fig. S1B, C). Notably, both necroptotic and apoptotic cells were enriched in the crypt region, possibly due to the enriched expression pattern of METTL3 in the crypts (Liu et al. 2023). Based on the cell location and LYZ co-staining, a substantial number of necroptotic and apoptotic cells were found in both stem cells and transient amplifying (TA) cells but not Paneth cells (Fig. 1A, B and Fig. S1D, E). Consistently, the transcriptional levels of genes associated with necroptosis and apoptosis were upregulated (Fig. 1C, D), which is confirmed by qPCR analysis (Fig. 1E). Immunoblotting further validated the activation of apoptotic and necroptotic pathways (caspase3, RIP3 and MLKL activation) in crypts and organoids derived from *Mettl3*-KO mice (Fig. 1F and Fig. S1F). Transmission electron microscopy also revealed subcellular features of cell death, including apoptotic body, necrosis-like swelling mitochondria and a low electronic condensation of the cytoplasm in *Mettl3*-KO crypt cells (Fig. 1G). Additionally, apoptotic and necroptotic signals were detected in the same epithelial cell as shown by p-MLKL and c-caspase3 co-staining (Fig. 1H). Further examination of genes related to apoptosis and necroptosis, such as *Casp8* and *Ripk1* (Christofferson et al. 2014; Dannappel et al. 2014; Guo et al. 2016), revealed reduced m^6^A modification and elevated gene expression upon *Mettl3*-KO (Fig. S1G). Together, these observations indicate that *Mettl3* depletion triggered extensive apoptosis and necroptosis of intestinal epithelial cells.

### Mettl3 deletion-induced cell death depends on RIPK1

**Fig. 2:**
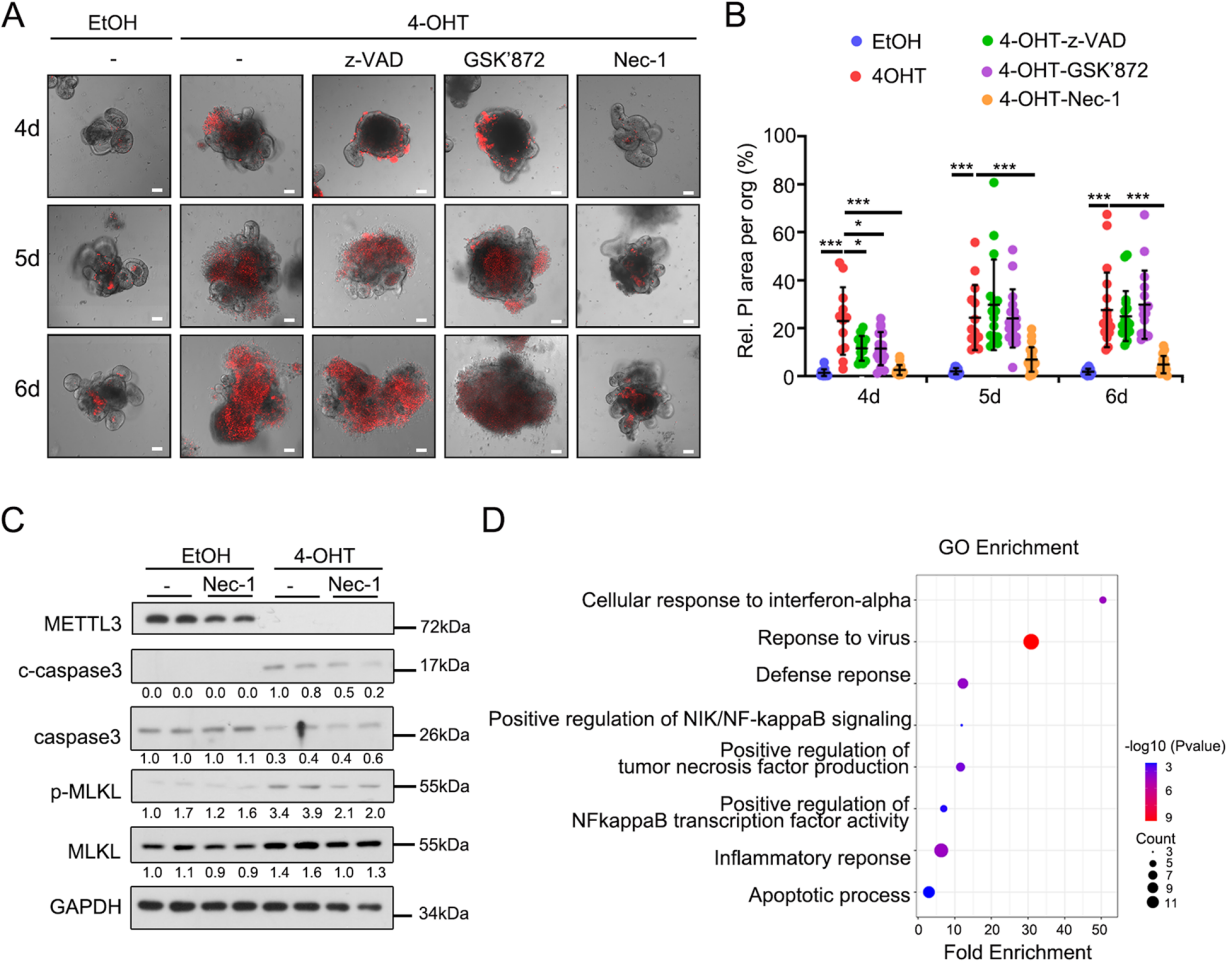
RIPK1 is required for *Mettl3* deficiency-induced cell death. **A**, **B** PI-staining was performed in the organoids derived from *Vil-CreERT2*;*Mettl3*^*fl/fl*^ mice and treated with EtOH or 4-OHT for 2 days in the presence of DMSO, Nec-1 (5µM), GSK872 (10µM) or z-VAD (10µM) for 4, 5, 6 days, as indicated by representative images (**A**) and statistical analysis (**B**) of the relative PI area per organoid (*n* ≥ 10 organoids). Data were from three independent experiments. **C** Immunoblotting and quantitative analyses of cell death-associated proteins in *Vil-CreERT2*;*Mettl3*^*fl/fl*^ derived organoids treated with EtOH or 4-OHT for 2 days in the presence of DMSO or Nec-1 for 5 days. GAPDH, loading control. **D** Functional enrichment analysis of downregulated genes (Nec-1/DMSO in 4-OHT group) in *Vil-CreERT2*;*Mettl3*^*fl/fl*^ organoids treated with 4-OHT for 2 days in the presence of DMSO or Nec-1 for 5 days. All the data represent mean ± SD. The data were analyzed by Two-way ANOVA (B). *P < 0.05, **P < 0.01 and ***P < 0.001. Scale bars: 50 μm (**A**)

To discern which cell death mode ultimately leads to death of *Mettl3*-KO epithelial cells, we tested the effect on organoids death of various cell death inhibitors, targeting apoptosis, necroptosis, and pyroptosis, respectively. The RIPK1 inhibitor necrostatin-1 (Nec-1) reduced cell death of *Mettl3*-KO organoids, although the pan-caspase inhibitor z-VAD-FMK (z-VAD), the RIPK3 inhibitor GSK'872, or the GSDMD inhibitors disulfiram and Ac-FLTD-CMK had no effect (Fig. 2A, B and Fig. S2A, B). Consistently, Nec-1 blocked *Mettl3* deletion-induced activation of caspase3, MLKL and RIPK1 (Fig. 2C and Fig. S2C) (Degterev et al. 2008), indicating Nec-1 might rescue cell death by impeding both necroptosis and apoptosis. Additionally, bulk RNA-seq analysis showed that Nec-1 treatment decreased the expression of cell death genes in *Mettl3*-KO organoids (Fig. S2D). Besides, Nec-1 restrained signaling pathways associated with cell death and inflammation in *Mettl3*-KO organoids, such as TNF production, NF-κB activity and the apoptotic process (Fig. 2D). These data together suggest that *Mettl3* could regulate apoptotic and necroptotic signaling via a RIPK1-dependent mechanism.

### Mettl3 regulates ATF3 mRNA stability

**Fig. 3:**
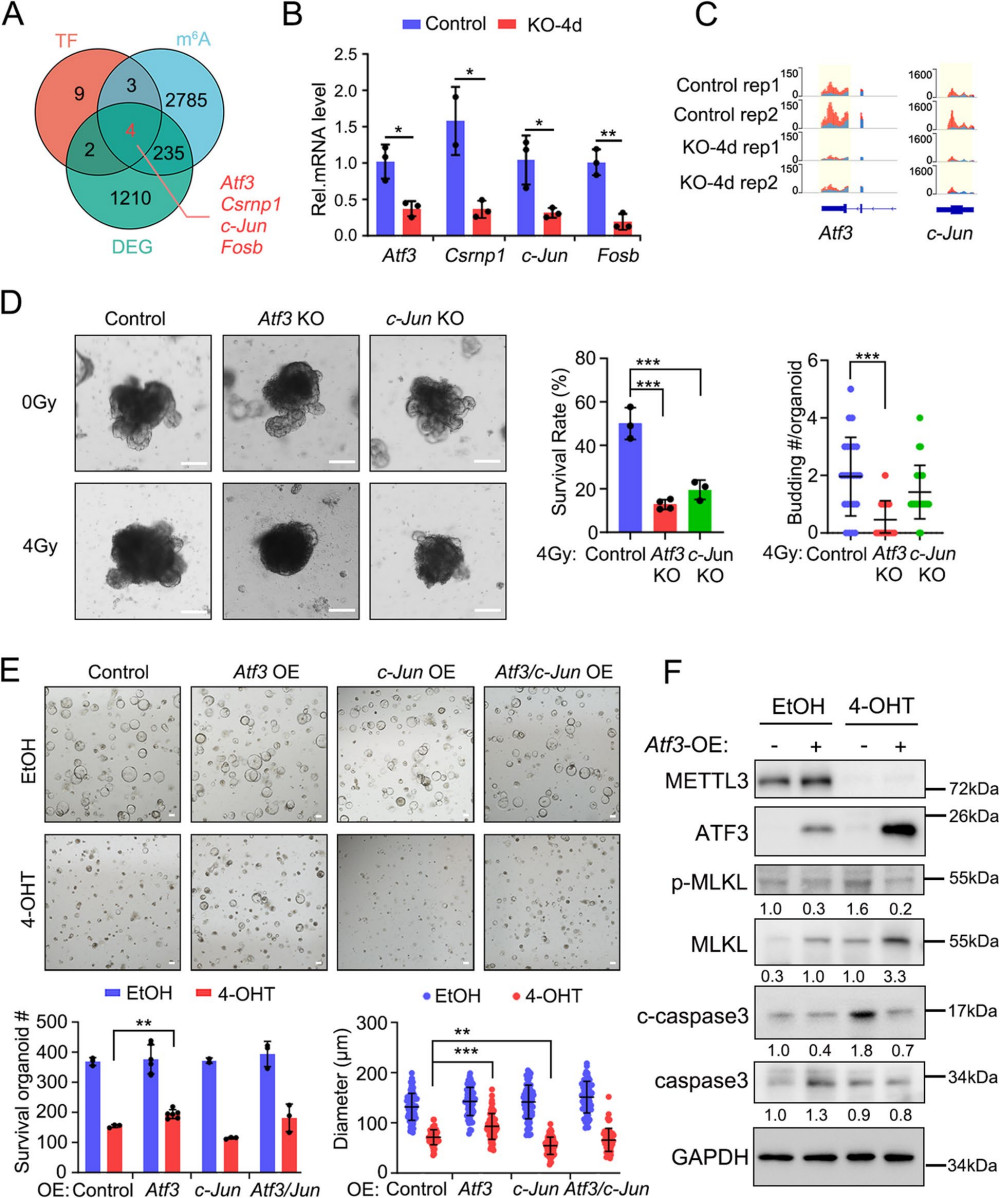
ATF3 regulates cell death in the intestinal epithelium. **A** Venn diagram depicting the overlap of predicted transcription factors that regulate cell death-related genes, genes exhibiting altered m^6^A modification, and differentially expressed genes in *Mettl3*-KO mice. **B** Expression of *Atf3*, *Csrnp1*, *Fosb* and *c-Jun* in FACS-sorted Lgr5^high^ cells from control and *Mettl3*-Lgr5-KO mice at 4 dpt, revealed by q-PCR. *n* = 3 mice per group, each dot represents one mouse. **C** Integrative Genomics Viewer (IGV) tracks displaying MeRIP-seq reads along the *Atf3* and *c-Jun* genes in Lgr5^high^ ISCs of control and *Mettl3*-KO mice. Blue reads were from input libraries and red reads from anti-m^6^A immunoprecipitation libraries. The Y axis represents the CPM (count per million) of genes. The yellow boxes of the tracks depict the positions of m^6^A peaks. **D** Representative morphology (left), survival rate and budding number (right) of organoids, which were derived from *Vil-CreERT2*;*Rosa26*^*loxp−stop−loxp−Cas9−EGFP*^ mice at 6 days post 0~4 Gy irradiation, and then infected with AAV to deplete *Atf3* or *c-Jun*. Data were from one of three independent experiments. **E** Representative morphology (upper), survival number and diameter (bottom) of organoids, which were derived from sorted RFP^+^ infected cells from *Vil-CreERT2*;*Mettl3*^*fl/fl*^ mice, and were infected with retrovirus expressing *Atf3*, *c-Jun*, or both *Atf3* and *c-Jun.* The cells were treated with EtOH or 4-OHT for 2 days and subjected for analysis at day 4. About 10,000 cells were seeded in each well for single cell culture. Data were from three independent experiments. **F** Immunoblotting and quantitative analyses of cell death-associated proteins in organoids derived from *Vil-CreERT2*;*Mettl3*^*fl/fl*^ mice, The organoids were treated with EtOH or 4-OHT for 2 days and then infected with control or *Atf3*-expressed lentivirus. GAPDH, loading control. All the data represent mean ± SD. The data were analyzed by One-way ANOVA (**D**) and Two-way ANOVA (**B**, **E**). *P < 0.05, **P < 0.01 and ***P < 0.001. Scale bars: 100 μm (**D**, **E**)

Given that METTL3 regulates apoptosis and necroptosis mainly through a RIPK1-dependent manner in IECs, we explored the mechanism how METTL3 regulates RIPK1-dependent cell death. To search for candidates regulated by *Mettl3*-mediated m^6^A modification, we integrated the RNA-seq, MeRIP-seq and single-cell RNA-seq (scRNA-seq) data for comprehensive analysis. As transcription regulation is mainly involved in altered m^6^A-methylated transcripts (Liu et al. 2023), we predicted upstream transcriptional factors by analyzing the differentially expressed genes associated with cell death using the chEA3 online tool (Fig. S3A). By combining the MeRIP-seq data, RNA-seq data and qPCR confirmation, we found that 4 TFs, *Atf3*, *Csrnp1*, *Fosb* (Liu et al. 2023) and *c-Jun*, exhibited reduced m^6^A methylation and low mRNA expression in *Mettl3* KO cells (Fig. 3A-C and Fig. S3B). *Csrnp1* transcript exhibited low expression in the intestinal epithelium (Fig. S3C), and *Fosb* mainly regulated stemness but not cell death (Liu et al. 2023).

Therefore, we focused on *Atf3* and *c-Jun*, and conducted knockout or overexpression assay to explore their function in organoids (Fig. S3D-F). Organoids subjected to in vitro irradiation conditions undergo organoid damage and cell apoptosis (Kim et al. 2022; Morral et al. 2024). Although *Atf3*-KO and *c-Jun*-KO organoids displayed normal morphology, upon irradiation, the survival rate and budding number of these organoids decreased, suggesting that *Atf3* and *c-Jun* play a role in suppressing cell death (Fig. 3D). Conversely, *Atf3-*overexpression partially restored the survival rate and spheroid size in *Mettl3*-KO organoids (Fig. 3E). Moreover, *Atf3*-overexpressing organoids showed reduced levels of cleaved-caspase3, p-MLKL, p-RIPK1 and p-RIPK3 compared to *Mettl3*-KO organoids (Fig. 3F and Fig. S3G), indicating that *Atf3* could inhibit RIPK1-dependent apoptosis and necroptosis. Meanwhile, the total protein level of RIPK3 and MLKL were slightly upregulated upon ATF3 overexpressed, consistent with previous study that ATF3 could regulate the expression of *Ripk3* and *Mlkl* (Inaba et al. 2023). In contrast, the organoids that overexpressed *c-Jun* exhibited the opposite effect (Fig. 3E). This may attribute to the dual function of *c-Jun* in both cell survival and cell death (Eferl and Wagner 2003).

### ATF3 upregulates cFLIP to suppress cell death

**Fig. 4:**
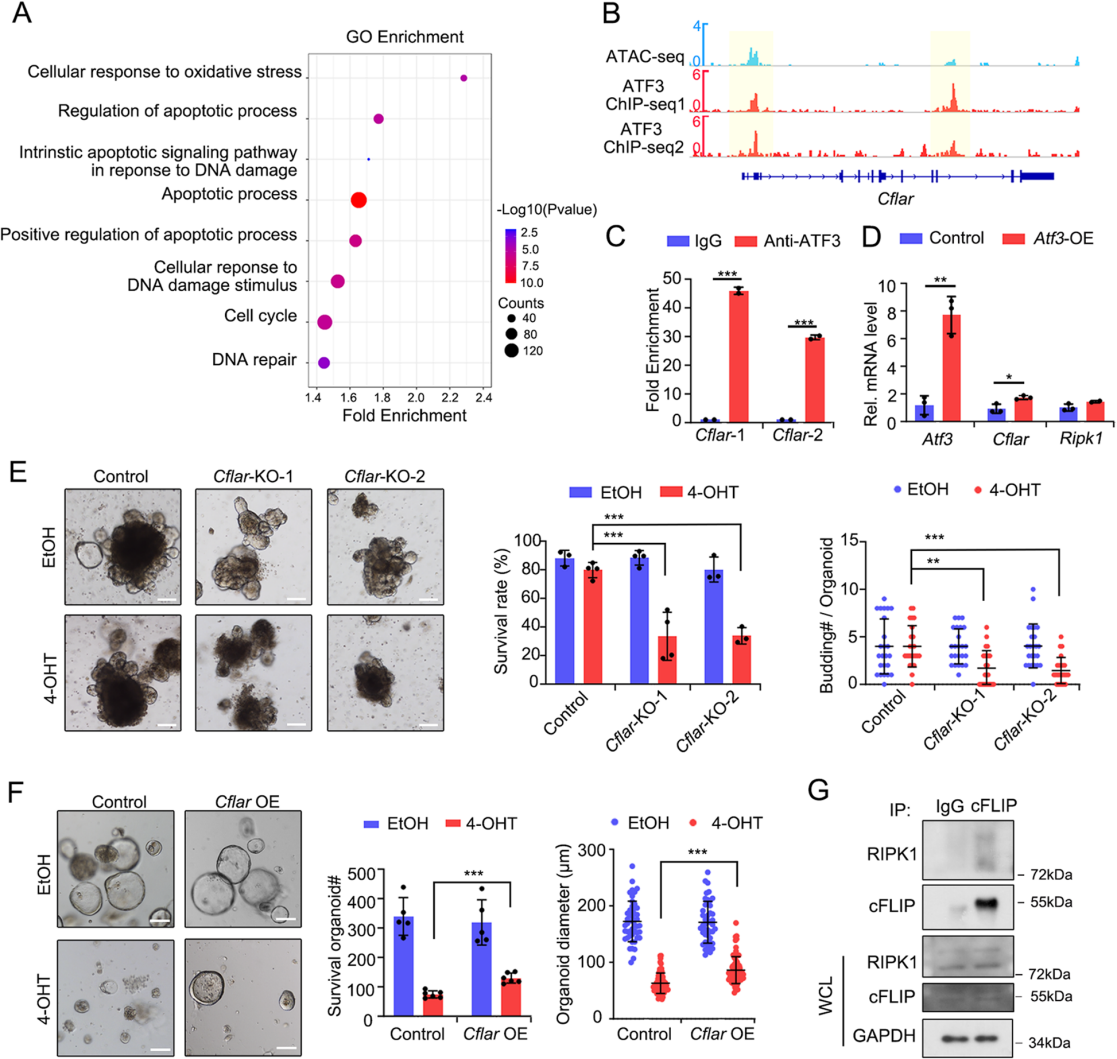
ATF3 regulates cell death in *Mettl3*-KO organoids via cFLIP. **A** GO functional enrichment analysis of ATF3 target genes. **B** ChIP-seq and ATAC-seq tracks of ATF3 binding to *Cflar* gene loci. **C** ChIP-qPCR analysis of ATF3 binding at regions near *Cflar* gene promoters in intestinal organoids. Fold change is shown relative to normal-IgG control. Data were from two independent experiments. **D** Expression of *Atf3*, *Cflar* and *Ripk1* in organoids derived from WT mice infected with control lentivirus (control) or Atf3-expressed lentivirus (*Atf3*-OE). Data were from three independent experiments. **E** Representative images of morphology (left), survival rate and budding number (right) of organoids derived from *Vil-CreERT2*;*Rosa26*^*loxp−stop−loxp−Cas9−EGFP*^ mice at 4 days, which were treated with EtOH or 4-OHT for 2 days and then infected with lentivirus to knock out *Cflar*. Data were from one of three independent experiments. **F** Representative morphology (left), survival number and diameter (right) of intestinal organoids derived from RFP^+^ infected cells sorted from *Vil-CreERT2*;*Mettl3*^*fl/fl*^ mice, which were infected with retrovirus expressing *Cflar.* The cells were treated with EtOH or 4-OHT for 2 days and subjected for analysis at day 4. About 10,000 cells were seeded in each well for single cell culture. Data were from one of three independent experiments. **G** Immunoblotting analyses of co-immunoprecipitation with IgG or cFLIP antibodies in organoids derived from the crypts of *Vil-CreERT2*;*Mettl3*^*fl/fl*^ mice, which were infected with cFLIP-expressing lentivirus. GAPDH, loading control. Data were from one of three independent experiments. All the data represent mean ± SD. The data were analyzed by Two-way ANOVA (**C**, **D**, **E**, **F**). *P < 0.05, **P < 0.01 and ***P < 0.001. Scale bars: 100 μm (**E**, **F**)

To further investigate the mechanism by which ATF3 regulates RIPK1-dependent cell death signaling in the intestinal epithelium, we performed ChIP-seq to identify ATF3 downstream targets. ATF3 binding was enriched in the DNA motif GG/AGGCGGG/A (Fig. S4A), and its target genes were implicated in the processes of apoptotic process and DNA damage (Fig. 4A). As ATF3 weakly bound to the *Ripk1* gene loci (Fig. S4B), by combining *Atf3* targets and *Mettl3*-KO-induced differentially expressed cell death genes, we identified *Cflar* (Fig. S4C), which encoded cFLIP (Cellular FLICE-like inhibitory protein), a suppressor of RIPK1-mediated cell death complex (Davidovich et al. 2023; Delanghe et al. 2020; Silke and Strasser 2013). Indeed, ATF3 bound to the *Cflar* gene, and the loci were correlated with the accessible chromatin, as indicated by assay for transposase-accessible chromatin with sequencing (ATAC-seq) analysis (Fig. 4B, C). The expression of *Cflar* was upregulated in *Atf3*-OE organoids and downregulated in *Mettl3*-KO ISCs and crypt cells (Fig. 4D and Fig. S4D, E), implying the *Cflar* was positively regulated by ATF3. More importantly, compared to wild-type organoids, *Cflar*-KO organoids exhibited a lower survival rate, fewer budding number and elevated c-caspase3 (Fig. 4E and Fig. S4F). Furthermore, *Cflar*-overexpression partially rescued the survival rate and spheroid size of *Mettl3*-KO organoids (Fig. 4F). Co-immunoprecipitation assay revealed cFLIP interacted with RIPK1 (Fig. 4G). Together, these data indicate that cFLIP, as a target of ATF3, interacts with RIPK1 and participates in *Mettl3* KO-induced cell death in the intestinal epithelium.

### Disruption of RIPK1 activity rescues *Mettl3 *KO-induced cell death

**Fig. 5:**
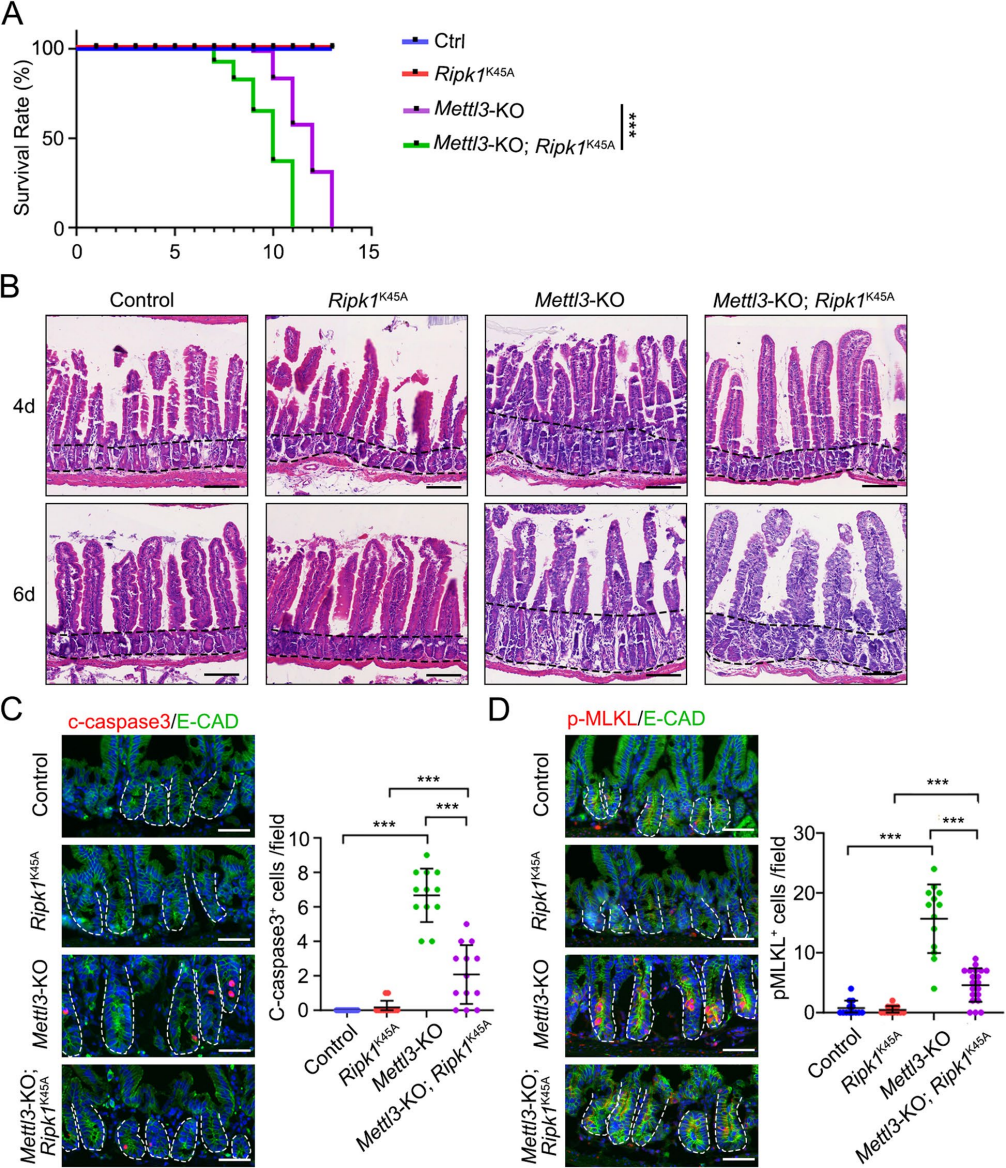
Genetic inactivation of RIPK1 attenuates apoptosis and necroptosis in *Mettl3* deficiency mice. **A** Survival plot for control (*Mettl3*^*fl/fl*^), *Ripk1*^*K45A*^, *Mettl3*-KO (*Vil-CreERT2*;*Mettl3*^*fl/fl*^) and *Mettl3*-KO;*Ripk1*^*K45A*^ (*Vil-CreERT2*;*Mettl3*^*fl/fl*^;*Ripk1*^*K45A*^) mice of 8~10 weeks old after injected with 20 mg/mL TAM for 4 days. (*n* ≥ 11 mice/group). **B** Representative H&E images of the jejunum from control, *Ripk1*^*K45A*^, *Mettl3*-KO and *Mettl3*-KO;*Ripk1*^*K45A*^ mice at the indicated time after TAM injection. *n* = 3 mice/group. The black dotted line represents crypt length. **C**, **D** Representative images and quantification of c-caspase 3^+^ cells (**C**) and p-MLKL^+^ cells (**D**) in jejunum crypts of control (*Mettl3*^*fl/fl*^), *Ripk1*^*K45A*^, *Mettl3*-KO (*Vil-CreERT2*;*Mettl3*^*fl/fl*^) and *Mettl3*-KO;*Ripk1*^*K45A*^ (*Vil-CreERT2*;*Mettl3*^*fl/fl*^;*Ripk1*^*K45A*^) mice at 4 dpt. *n* = 3 mice/group. All the data represent mean ± SD. The data were analyzed by Log-rank test (**A**) and One-way ANOVA (**C**, **D**). *P < 0.05, **P < 0.01 and ***P < 0.001. Scale bars: 100 μm (**B**), 50 μm (**C**, **D**). Nuclei were counter-stained with DAPI

To illustrate the function of *Ripk1* in *Mettl3*-KO mice, we knocked down *Ripk1* in organoids derived from *Vil-CreERT2*;*Mettl3*^*fl/fl*^ mice. Since *Ripk1* knockdown partially rescued cell survival and increased budding number in *Mettl3*-KO organoids (Fig. S5A), we assessed whether genetic inactivation of RIPK1 prevents intestinal epithelial cell death in *Mettl3*-deficient mice. We crossed *Vil-CreERT2*;*Mettl3*^*fl/fl*^ mice with *Ripk1*^*K45A*^-knockin mice, which harbor a RIPK1 kinase-dead K45A mutation (Fig. S5B) (Liu et al. 2017). Genetic inactivation of RIPK1 delayed the death time from 7~11 days to 10~13 days (Fig. 5A). Additionally, the crypt elongation and the epithelial structure were partially restored (Fig. 5B and Fig S5C). Moreover, *Mettl3*-KO mice harboring RIPK1(K45A) exhibited less c-caspase 3 and p-MLKL cells compared to *Mettl3*-KO mice (Fig. 5C, D and Fig. S5D, E). Therefore, these findings suggest that RIPK1 plays a critical role in *Mettl3* KO-induced intestinal epithelial cell death.

## Discussion

**Fig. 6:**
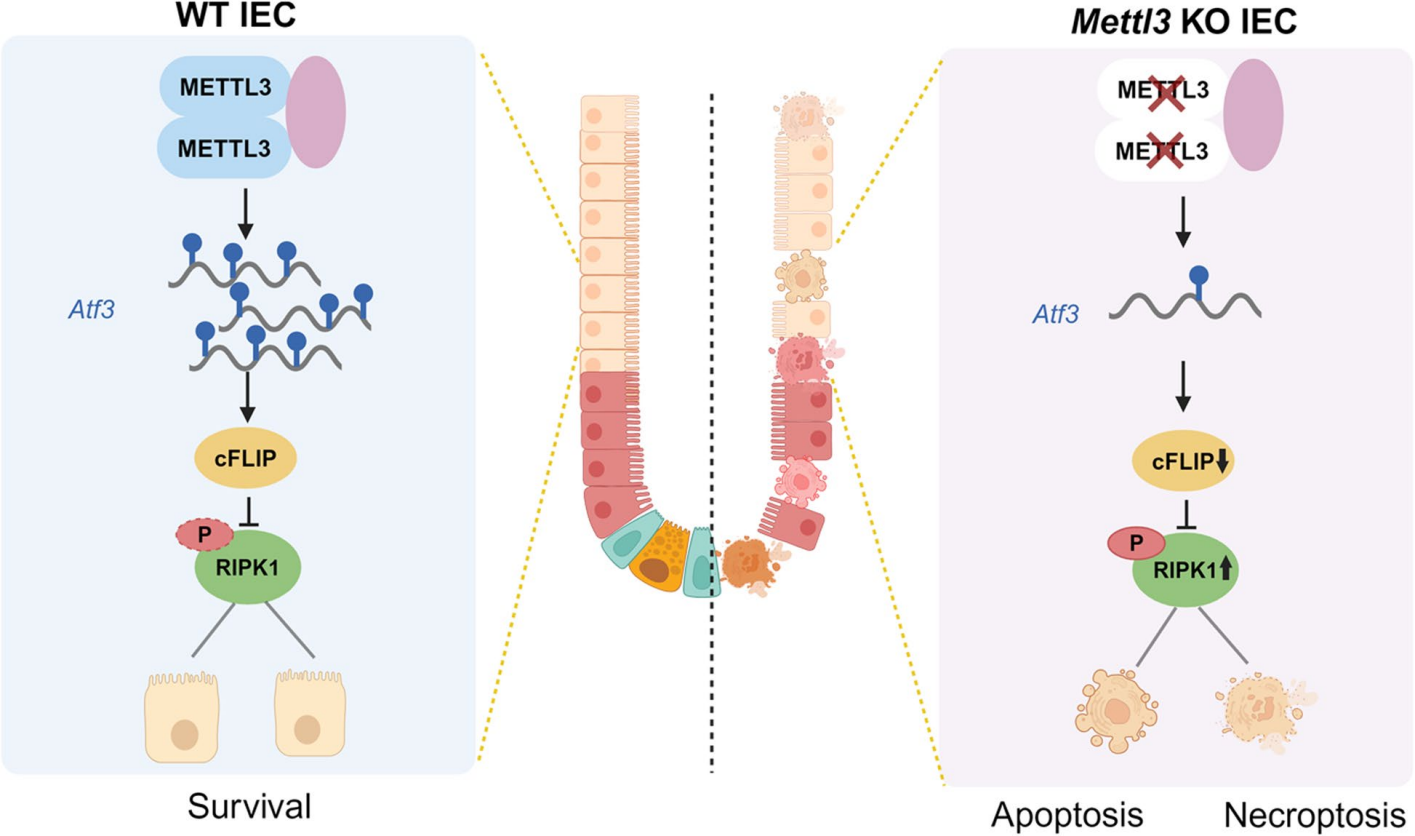
A Schematic overview depicting the role of METTL3 in regulating apoptosis and necroptosis in the intestinal epithelium. In intestinal epithelial cells, ATF3 is regulated by METTL3-mediated m^6^A modification. ATF3 binds to and regulates the expression of *Cflar*, the gene encoding the anti-cell death protein cFLIP, which inhibits RIPK1-mediated apoptosis and necroptosis. *Mettl3* deficiency leads to its low expression, high RIPK1 activity and consequently cell death

It has been reported that m^6^A modification plays an important role in regulation of stemness of ISCs and regeneration of the intestinal epithelium (Han et al. 2020; Liu et al. 2023). In this study, we found that METTL3-mediated m^6^A modification is crucial for regulating cell death of the small intestinal epithelial cells, thereby ensuring the maintenance of intestinal epithelial homeostasis (Fig. 6), which is in accordance with the role of m^6^A modification in preserving colon epithelial cell survival by targeting apoptosis (Du et al. 2022; Zhang et al. 2022). Interestingly, we observed that *Mettl3*-deficiency could induce both apoptosis and necroptosis in small intestinal epithelial cells. Moreover, we found that both apoptosis and necroptosis markers were in a minimal fraction of the same intestinal epithelial cells, indicating that these cells may even undergo concurrent apoptosis and necroptosis. It is worthy of further investigation as to how apoptosis and necroptosis take place in single cell. Inhibition of RIPK1 could prevent *Mettl3*-KO-induced cell death, whereas the pan-caspase inhibitor or RIPK3 inhibitor did not, demonstrating the essential role of RIPK1 in *Mettl3*-KO induced cell death. That is consistent with the previous report that RIPK1 could mediate apoptosis and necroptosis via TNFR1 signaling cascades (Xu et al. 2021).

IECs have been reported to undergo different types of cell death (Patankar and Becker 2020; Subramanian et al. 2020). TAK1 and caspase 8 are identified as pivotal regulators of IEC apoptosis versus necroptosis programs downstream of TNFR1 (Fritsch et al. 2019; Geng et al. 2017; Newton et al. 2019). TAK1-activated NF-κB signaling is required for cell survival (Kajino-Sakamoto et al. 2008; Nenci et al. 2007; Pasparakis 2009; Steinbrecher et al. 2008; Sträter and Möller 2000). Their deletion triggers the apoptotic cascade through the recruitment of FADD and caspase 8 to TNF receptor complex (Patankar and Becker 2020). Ablation of FADD or caspase 8 in IECs leads to non-apoptotic cell death and spontaneous gut inflammation, which can be reversed by further knockout of RIPK3 (Günther et al. 2011; Welz et al. 2011). These results indicate that FADD or caspase 8 deficiency causes intestinal inflammation by sensitizing IECs to RIPK3-dependent necroptosis. TAK1 has also been shown to enhance FLIP degradation, facilitating the formation of the RIPK1-FADD-caspase 8 complex, leading to apoptosis (Guo et al. 2016). Moreover, an optimal level of caspase 8 activity is essential for maintaining IEC stability: high activation initiates apoptosis while low activity leads to necroptosis (Ruder et al. 2018). Besides METTL3-ATF3-cFLIP-RIPK1 axis, we also observed that *Mettl3* deletion induces an increased expression of *Ripk1* and *Casp8* (Fig S1 F, G). The upregulation of total *Casp8* and *Ripk1* expression resulting from *Mettl3* knockout may induce increased apoptosis and partially inhibit necroptosis. However, necroptosis still occurs in the intestinal epithelial cells by combining the effects of the METTL3-ATF3-cFLIP-RIPK1 axis. Moreover, METTL3 could regulate *Ripk1* gene expression through m^6^A modifications, as well as inhibit RIPK1 activation via the METTL3-ATF3-cFLIP-RIPK1 axis.

We identified two transcriptional factors regulated by METTL3-mediated m^6^A modification, *Atf3* and *c-Jun*, which belong to the members of the activator protein-1 (AP-1) family. AP-1 transcription factors regulate cell proliferation, cell survival and cell death (Shaulian and Karin 2002). ATF3 was shown to maintain intestinal barrier regeneration and IEC survival (Yang et al. 2009; Zhou et al. 2017), while it also promotes IEC apoptosis by working with p53 in Crohn’s disease (Gu et al. 2018). Here, we demonstrated that *Mettl3*-mediated m^6^A modification is essential for *Atf3* mRNA stability. We further showed that ATF3 prevents apoptosis and necroptosis by upregulating cFLIP, a suppressor of RIPK1. Therefore, ATF3 plays a crucial role in safeguarding intestinal barrier under stress. *c-Jun* knockout also enhanced cell death in intestinal organoids, supporting its role in mediating *Mettl3*-KO-induced cell death. However, *c-Jun* overexpression also triggered cell death in organoids, which is consistent with the early report (Bossy-Wetzel et al. 1997). These data emphasize the context-dependent role of *c-Jun* in cell proliferation and death (Shaulian and Karin 2001, 2002), which need further investigation.

## Materials and methods

### Mice

*Mettl3*^*fl/fl*^ mice were from Dr. Wei Li (Institute of Zoology, CAS) (Xu et al. 2017), and *Vil-CreERT2* mice were a gift from Dr. Sylvie Robine (Institut Curie-CNRS) (Ireland et al. 2004). *Ripk1*^*K45A*^ mice from Dr. Xiao-Dong Wang (National Institute of Biological Sciences) (Liu et al. 2017). Above mice were crossed to generate *Vil-CreERT2*;*Mettl3*^*fl/fl*^ mice and *Vil-CreERT2*;*Mettl3*^*fl/fl*^;*Ripk1*^*K45A*^ mice. Male mice aged 2~4 months were used in this study. All animal studies were conducted in accordance with the relevant guidelines and under the approval of the Institutional Animal Care and Use Committee of Tsinghua University.

For Cre induction in mice intestinal epithelium, *Vil-CreERT2*;*Mettl3*^*fl/fl*^ mice and *Vil-CreERT2*;*Mettl3*^*fl/fl*^;*Ripk1*^*K45A*^ mice were intraperitoneally injected at a dose of 100 mg/kg body mass tamoxifen (Sigma, T5649) in corn oil for 4 consecutive days. *Mettl3*^*fl/fl*^ mice and *Mettl3*^*fl/fl*^;*Ripk1*^*K45A*^ mice were treated with an equal amount of tamoxifen as corresponding control group.

### Isolation of intestinal crypts and organoid culture

Mouse small intestinal crypts were isolated and cultured following the established procedures (Qi et al. 2017). Briefly, the mouse intestine was longitudinally cut and washed with cold PBS. After carefully removing villi, small intestine pieces were incubated in PBS containing 2 mM EDTA for 30 min on ice. The pieces were then vigorously suspended in cold PBS, and the mixture was filtered through a 70 μm cell strainer (BD Biosciences). The crypt fraction was enriched by centrifugation. Then the crypts were embedded in Matrigel (Corning, #356,231) and seeded on 24-well plate. After polymerization, the crypts were cultured in ENR medium (Advanced DMEM/F12 containing EGF (50 ng /mL, Invitrogen), Noggin (100 ng /mL, OrganRegen) and R-spondin1 (500 ng /mL, OrganRegen), Penicillin/Streptomycin, GlutaMAX-I, N2, B27 and N-acetylcysteine (Sigma-Aldrich). For passaging, the organoids were suspended with cold PBS, centrifuged and then re-embedded in fresh Matrigel before seeding on a plate.

To induce *Mettl3* ablation in the organoids derived from *Vil-CreERT2*;*Mettl3*^*fl/fl*^ mice, 500 nM 4-Hydroxytamoxifen (4-OHT) was added to the ENR culture medium for 2 days. For spheroid colony formation, alive RFP^+^ cells (which were infected with virus) were sorted by flow cytometry, then embedded in fresh Matrigel, cultured in expansion medium (ENR culture medium plus 2.5 µΜ CHIR-99,021, 10% Wnt-3a conditional medium and 6.67 µM blebbistatin).

### Virus production and organoid infection

Retrovirus and lentivirus were used for overexpressing *Atf3*, *c-Jun* and *Cflar* in the organoids, the corresponding plasmids were pMSCV-loxp-dsRed-loxp-eGFP-Puro-WPRE (Addgene, 32,702) and pLVX-IRES-Puro (Clontech, 632,183). Mouse *Atf3*, *c-Jun* and *Cflar* cDNAs were constructed into these two vectors for further overexpression experiment.

Retrovirus, lentivirus and recombinant adeno-associated virus (AAV) were produced as previously described (Koo et al. 2011; Li et al. 2021; Liu et al. 2021). Before virus infection, organoids underwent a two-day culture with expansion medium supplemented with 10 mM nicotinamide. Subsequently, the organoids were digested to cell pellet using TrypLE (Gibco, 12,604,021). The cell pellet was re-suspended in expansion medium supplemented with 10 µg/mL polybrene (Macgene, MC032), and mixed with the virus. The cell-virus mixture (250 µL) in expansion medium plus polybrene was then added to pre-solidified Matrigel and incubated overnight at 37 °C. Next day, the medium was removed, and the virus was washed with warm PBS. Following this, a 10 µL Matrigel overlay was applied, and the organoids were cultured in expansion medium.

*Vil-CreERT2*;*Mettl3*^*fl/fl*^ organoids were prepared for retrovirus and lentivirus infection, and the culture medium was replaced with ENR supplemented with 2 µg/mL puromycin at day 2 post-infection. Once the organoids exhibited normal growth under puromycin selection, both protein expression and *Mettl3*-KO was induced using 4-OHT (0.5µM, Sigma, H7904). *Vil-CreERT2*;*Rosa26*^*loxp−stop−loxp−Cas9−EGFP*^ organoids were prepared for recombinant AAV infection and pretreated with 4-OHT to induce Cas9 expression. The sgRNA sequences are listed in Supplementary Table S1.

### Image acquisition and analysis of organoid cell death

For cell death inhibitor treatment, *Vil-CreERT2*;*Mettl3*^*fl/fl*^ organoids were treated with EtOH or 4-OHT for 2 days. In the 4-OHT treatment group, DMSO or 10 µM z-VAD (Selleck, S7023), 10 µM GSK’872 (Selleck, S8465), 10 µM Necrostatin-1 (Selleck, S8037), 5 µM Disulfiram (MCE, HYB0240), 10 µM Ac-FLTD-CMK (MCE, HY-111,675), were added to the culture medium. At day 4 post-treatment, 1 µg/mL propidium iodide (PI) dye was added and incubated for 30 min. Subsequently, on the 4, 5, 6 days post-treatment, fluorescent microscopy was employed to observe the PI staining within the organoids.

### Tyramide signal amplification (TSA)-mediated immunofluorescence and H&E staining

A fluorescence kit based on TSA technology was employed following the manufacturer’s instruction (Recordbio Biological Technology, RC0086-23R). Intestinal tissues were fixed in 10% formalin at room temperature for 2 h, dehydrated in 20% sucrose solution overnight, and subsequently embedded for cryo-sections. For antigen retrieval, sections underwent boiling at 100 °C for 10 min in a pH 6.0 citric acid buffer solution within a microwave-enabled retrieval chamber. To inhibit endogenous peroxidase activity, sections were treated with a 3% hydrogen peroxide solution at room temperature for 15 min. 3% bovine serum albumin was applied to block non-specific signals for 30 min at room temperature. The sections were then incubated overnight at 4 °C with the primary antibody. Next day, the sections were incubated with horseradish peroxidase-conjugated secondary antibody (anti-mouse or anti-rabbit) for 50 min at room temperature. Following the incubation, fluorescence labeling was achieved by treating the sections with TYR520 fluorescent dye for 10~15 min. For cleaved-caspase 3 (1:300, CST, 9664S) and p-MLKL (1:1600, Abcam, ab196436) co-staining, repeat previous steps from the antigen retrieval, and switch to TYR570 fluorescent dye for p-MLKL. After incubation for fluorescent dye, DAPI was staining for another 15 min.

For hematoxylin-eosin staining, the intestinal tissues were fixed in 4% paraformaldehyde at room temperature overnight, then sequentially in 70%, 80%, 90%, 95%, and 100% ethanol, followed by further dehydration in xylene. The sample was placed in paraffin, immersed for 2 h, and subsequently embedded to create paraffin-sections. Paraffin-sections were automatically stained using a stainer integrated workstation (Leica ST5020-CV5030), the time of hematoxylin staining was 30s.

### Transmission electron microscopy (TEM)

The mice intestine was longitudinally opened and cut into approximately 5 mm pieces, followed by fixation in 2.5% glutaraldehyde in PBS immediately. After embedding in Epon Araldite, ultrathin sections were sliced along the crypt-villus axis and analyzed using a Hitachi HT-7800 TEM.

### Flow cytometry

Organoids infected with retrovirus were incubated in TrypLE (Gibco, 12,604,021) for 20 min at 37 ^o^C to obtain single-cell suspension. The dissociated cells were subsequently filtered through a 40 μm cell strainer (BD), staining with 4’,6-Diamidino-2-Phenylindole (DAPI, Invitrogen, D1306). Infected cells (DAPI^−^RFP^+^) were sorted using flow cytometry (MoFlo Astrios EQ, Beckman).

### Quantitative real-time PCR (qRT–PCR)

Total RNA was extracted from tissues or organoids using the RaPure Total RNA Kit (Magen, R4011), and cDNA was synthesized using cDNA Synthesis SuperMix (Novoprotein, E047-01A). qRT–PCR was conducted in triplicates on a LightCycler 480 (Roche) using NovoStart^®^SYBR qPCR SuperMix Plus (Novoprotein, E096-01A) with *Gapdh* as the reference gene. Data were analyzed according to the 2^−ΔΔCT^ method. Primer sequences are listed in Table S2.

### Immunoblotting

Protein lysates were prepared from intestinal epithelial crypts or organoids. Cells were lysed in RIPA buffer (Beyotime, P0013B) with protease inhibitors (Roche, 04693132001), PMSF (Beyotime, ST506) and phosphatase inhibitor (Solarbio, P1260). 40 µg of total protein was separated with 10% SDS-PAGE gel under denaturing conditions and were transferred to nitrocellulose membranes (PALL, 66,485). Following blocking, the membranes were incubated with primary antibodies and subsequently with secondary anti-rabbit or anti-mouse conjugated antibodies. Relative protein intensities were calculated by Image Lab software (Version 6.1), the relative level of total proteins was normalized to GAPDH and the relative level of phosphorylated proteins was normalized to the corresponding total protein.

The following primary antibodies were used: rabbit anti-METTL3 (1:1000, Abcam, ab195352), ATF3 (Abcam, ab207434), c-JUN (Abcam, ab32137), MLKL (1:1000, Abcam, ab172868), p-MLKL (1:1000, Abcam, ab196436), caspase-3 (1:1000, 350,192, Zenbio), cleaved caspase-3 (1:1000, CST, 9664S), cFLIP (1:1000, CST, 56,343S), p-RIPK3 (1:500, CST, 93,654), RIPK3 (1:1000, abcam, ab62344), p-RIPK1 (1:500, CST, 65,746), RIPK1 (1:1000, Bioss, bs-5805R), mouse anti-Tubulin (1:1000, MBL, PM054-7) and GAPDH (1:1000, MBL, M171-7).

### Immunoprecipitation

Co-immunoprecipitation conducted using Dynabeads Protein A/G immunoprecipitation Kit (10007D, Invitrogen) following the manufacturer’s instruction. For each sample, 10 µL Dynabeads Protein A and 10 µL Dynabeads Protein G were transferred to a low-binding tube, placed on a magnet, and the supernatant was removed. The beads were then resuspended in 200 µL Binding & Washing Buffer containing 1 µg of anti-rabbit-IgG (CST, 7074) or rabbit anti-cFLIP (CST, 56,343S) per sample, followed by a 4-hr incubation with rotation at 4 °C. The Dynabeads-antibody complex was separated with a magnet and washed using 200 µL Ab Binding & Washing Buffer. Lentivirus-infected organoids, either control or overexpressing *Cflip*, were lysed in 300 µL ice-cold IP lysis buffer (10 mM Tris-HCl, pH 7.5, 100 mM NaCl, 0.5% NP-40, 1 mM EDTA, 1mM PMSF, 50 mM NaF, 2 mM Na_3_VO_4_, protease inhibitor cocktails (04693132001, Roche)) for 1 h. After centrifugation at 13,000 rpm for 10 min. 30 µL soluble supernatants were harvested as input after preclearing, and the remaining soluble supernatants were immunoprecipitated with corresponding antibody binding Dynabeads with rotation overnight at 4 ℃. The immunoprecipitants were washed three times with PBS. The Dynabeads-antibody-antigen complex was gently resuspended in 30 µL loading buffer and analyzed by western blot.

### RNA-seq

Total RNA was extracted with RNeasy Mini Kit (QIAGEN) according to the manufacturer’s instruction. Bulk RNA-seq was performed using the Illumina Hiseq X Ten platform and analyzed as previously described (Liu et al. 2023).

### CUT&Tag ChIP-seq

Alive lentivirus-infected organoids overexpressing *Atf3* were sorted and collected into 500 uL PBS, cells conducted ChIP-seq following the NovoNGS CUT&Tag 3.0 High-Sensitivity Kit (Novoprotein, H259-YH01) and the lysates were incubated with anti-ATF3 antibody (1:100, Abcam, ab207434) at 4 ℃ overnight. DNA extracted using Tagment DNA Extract Beads was used as a templet, and amplification was performed with 2X KAPA HIFI HotStart polymerase (KAPA, KK2601). The library was purified with 1.4X AMPure beads and sequenced with the Illumina Novaseq 6000.

### ChIP-seq analysis

HISAT2 was used to align the sequences to the mouse genome and generate bam files. PCR duplicates were removed using Picard tools, and Deeptools (3.3.1) bamCoverage (CPM normalized and extended reads) was applied to generate bigwig files from bam files. MACS2 (v2.2.5) conducted peak calling and generated bed files from aligned reads. HOMER (v4.10.0) annotatePeaks.pl was used for peak annotation. Visualization of the binding peaks from bigwig files was performed using IGV (2.6.2). The motif of ATF3 was identified using findMotifsGenome.pl from HOMER.

### Gene ontology (GO) analysis

Genes meeting the criteria of *P* < 0.05 and |log_2_FC|≥1 were selected for Gene ontology (GO) analysis. The analysis was conducted using the web tool DAVID (http://david.adcc.ncifcrf.gov/), and GO terms with *P* < 0.05 were considered statistically significant. Venn diagrams were created using online venn diagram (https://bioinformatics.psb.ugent.be/webtools/Venn//).

### Methylated RNA immunoprecipitation (MeRIP)-seq, ATAC-seq and Single cell RNA sequencing (scRNA-seq) analysis

A dataset (GSE186917) of control and *Mettl3*-KO was employed in our research (Liu et al. 2023). We conducted visualizations of m^6^A level and the expression profile of *Casp8*, *Ripk1*, *Atf3*, *c-Jun*, *Csrnp1 and Fosb* using the original processing results obtained from MeRIP-seq and scRNA-seq data of control and *Mettl3*-KO mice (GSE186917). Additionally, the binding peak of *Cflip* was visualized based on the ATAC-seq data.

### Statistics

All experiments were independently repeated with at least three biological replicates. Statistical analysis was performed with Graphpad Prism (version 10). Data shown in graphs represent mean ± SD as indicated in the figure legends. Unpaired two-tailed t-test, one-way ANOVA, and two-way ANOVA analysis was used to compare differences as indicated in the figure legends.

### Supplementary Information

Additional file 1: Supplemental Information. Fig. S1. Ablation of *Mettl3* induces cell death in the intestinal epithelium. Fig. S2. Inhibition of RIPK1 activity mitigates cell death in *Mettl3*-KO organoids. Fig. S3. Identification of transcriptional factors that regulate cell death in the intestinal epithelium. Fig. S4. Identification of ATF3 target genes that regulate cell death in *Mettl3*-KO organoids. Fig. S5. RIPK1 inactivation alleviates cell death in *Mettl3*-KO organoids and mice.

Additional file 2: Supplementary Table 1: sgRNA sequences.

Additional file 3: Supplementary Table 2: qPCR primer sequences.

## Data Availability

The RNA-seq and ChIP-seq data generated in this study are publicly available through the Gene Expression Omnibus with the accession code GSE262282 (cut tag ChIP-seq and RNA-seq). The scRNA-seq data, m^6^A-seq data and bulk RNA-seq data (GSE186917) was used to analyze the expression pattern of *Atf3*, *c-Jun*, *Csrnp1 and Fosb*, and m^6^A modification and expression level of *Casp8* and *Ripk1* (Liu et al. 2023). All codes that the main steps of the analysis and data are available from the corresponding author under request.

## Abbreviations

IECs: Intestinal epithelial cells
ISCs: Intestinal stem cells
IBD: Inflammatory bowel diseases
RIPK: Receptor-interacting protein kinase
MLKL: Mixed-lineage kinase domain-like protein
m^6^A: N6-adenomethylation
METTL3: Methyltransferase-like 3
METTL14: Methyltransferase-like 14
FTO: Fat mass and obesity-associated protein
ALKBH5: AlkB homolog 5
GO: Gene ontology
TA: Transient amplifying
Nec-1: Necrostatin-1
z-VAD: z-VAD-FMK
scRNA-seq: Single-cell RNA sequencing
cFLIP: Cellular FLICE-like inhibitory protein
ATAC-seq: Assay for transposase-accessible chromatin with sequencing
AP-1: Activator protein-1
4-OHT: 4-hydroxytamoxifen
AAV: Adeno-associated virus
TSA: Tyramide signal amplification
TEM: Transmission electron microscopy
DAPI: 4’,6-Diamidino-2-Phenylindole
MeRIP-seq: Methylated RNA immunoprecipitation sequencing
