# Supplementary material for: METTL3 restricts RIPK1-dependent cell death via the ATF3-cFLIP axis in the intestinal epithelium
Source: Cell Regen. 2024 Aug 2;13:14. doi: 10.1186/s13619-024-00197-8 (PMC11297012; doi:10.1186/s13619-024-00197-8)
Supplement: Supplementary file 1 — Additional file 1: Supplemental Information. Fig. S1. Ablation of Mettl3 induces cell death in the intestinal epithelium. Fig. S2. Inhibition of RIPK1 activity mitigates cell death in Mettl3-KO organoids. Fig. S3. Identification of transcriptional factors that regulate cell death in the intestinal epithelium. Fig. S4. Identification of ATF3 target genes that regulate cell death in Mettl3-KO organoids. Fig. S5. RIPK1 inactivation alleviates cell death in Mettl3-KO organoids and mice. [file 13619_2024_197_MOESM1_ESM.docx]

**Supplemental Information**

**METTL3** **restricts RIPK1-dependent cell death via the ATF3-cFLIP axis in the intestinal epithelium**

Meimei Huang^1,3*^, Xiaodan Wang^1,*^, Mengxian Zhang^1,^, Yuan Liu^1,#^, Ye-Guang Chen^1,2,3,#^

**Figures S1-5.**

**Tables S1-2.**

**Fig. S1**

**
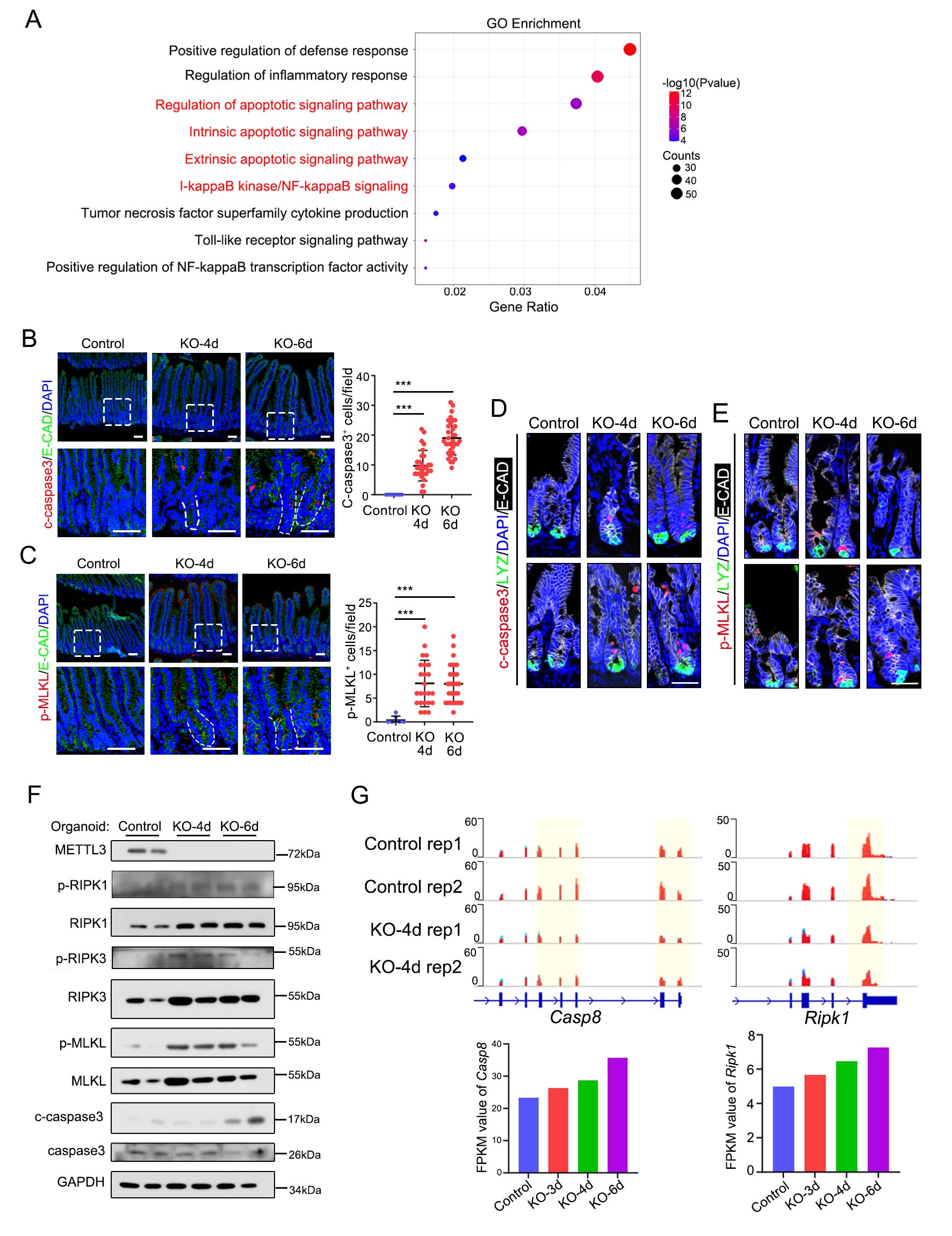
**

**Fig. S1** **Ablation of *Mettl3* induces cell death in the intestinal epithelium. (A)** Functional enrichment analysis of upregulated genes in ISCs of *Mettl3*-KO (*Vil-CreERT2;Mettl3^fl/fl^*) mice at 6 days after tamoxifen injection. **(B, C)** Representative images (left) and quantification (right) of c-caspase 3^+^ cells (B) and p-MLKL^+^ cells (C) in jejunum epithelium of control and *Mettl3*-KO mice at the indicated time after tamoxifen injection. n=3 mice/group. **(D, E)** Representative images of immunofluorescence co-staining of Paneth cell markers LYZ with c-caspase 3^+^ cells (D) and p-MLKL^+^ cells (E) in jejunum epithelium of control and Mettl3-KO mice at the indicated time after tamoxifen injection. n=3 mice/group. **(F)** Immunoblotting analysis of cell death-associated protein expression in organoids derived from *Vil-CreERT2*;*Mettl3^fl/fl^* mice at the indicated time after EtOH or 4-OHT treatment. GAPDH, loading control. **(G)** Integrative Genomics Viewer (IGV) tracks displaying MeRIP-seq reads along the *Casp8* genes and *Ripk1* gene in Lgr5^high^ ISCs of control and *Mettl3*-KO mice (Upper panel). Blue reads were from input libraries and red reads from anti-m^6^A immunoprecipitation libraries. The Y axis represents the CPM (count per million) of genes. The yellow boxes of the tracks depict the positions of m^6^A peaks. FPKM value of the *Casp8* gene and *Ripk1* gene in Lgr5^+^ intestinal stem cells of control and *Mettl3*-KO mice at day 3, 4, 6 post tamoxifen injection, based on bulk RNA-seq (Bottom panel). All the data represent mean±SD. The data were analyzed by One-way ANOVA (B, C). *P<0.05, **P<0.01 and ***P<0.001. Scale bars: 50 μm (B, C, D, E). Nuclei were counter-stained with DAPI.

**Fig. S2**


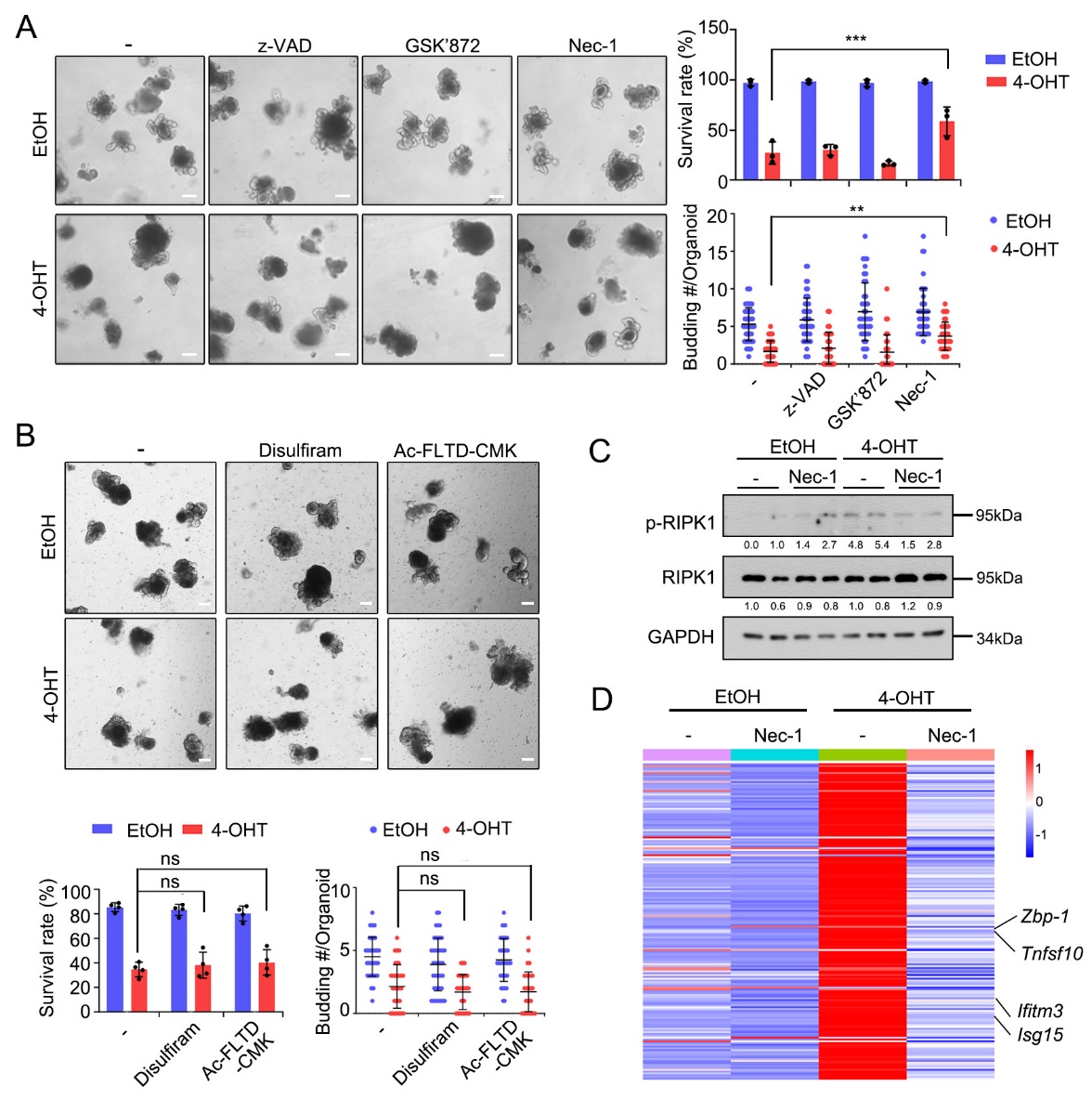


**Fig. S2 Inhibition of RIPK1 activity mitigates cell death in *Mettl3*-KO organoids. (A)** Representative morphology (left), survival rate and budding number (right) of intestinal organoids derived from *Vil-CreERT2;Mettl3^fl/fl^* mice and treated with EtOH or 4-OHT for 2 days in the presence of DMSO, Nec-1 (5μM), GSK'872 (10μM) or z-VAD (10μM) for 5 days. Data were from one of three independent experiments. **(B)** Representative morphology (upper), survival rate and budding number (bottom) of intestinal organoids derived from *Vil-CreERT2;Mettl3^fl/fl^* mice and treated with EtOH or 4-OHT for 2 days in the presence of DMSO, Disulfiram (5μM), Ac-FLTD-CMK (10μM) for 5 days. Data were from one of three independent experiments. **(C)** Immunoblotting and quantitative analyses of p-RIPK1 and RIPK1 in *Vil-CreERT2*;*Mettl3^fl/fl^* derived organoids treated with EtOH or 4-OHT for 2 days in the presence of DMSO or Nec-1 for 5 days. GAPDH, loading control. Each group contains two different samples, and each lane represents one sample. **(D)** Expression heatmap of downregulated genes (Nec-1/DMSO in 4-OHT group) in organoids derived from *Vil-CreERT2;Mettl3^fl/fl^* and treated with EtOH or 4-OHT for 2 days in the presence of DMSO or Nec-1 for 5 days. All the data represent mean±SD. The data were analyzed by Two-way ANOVA (A, B). *P<0.05, **P<0.01 and ***P<0.001. Scale bars: 100 μm (A, B).

**Fig. S3**


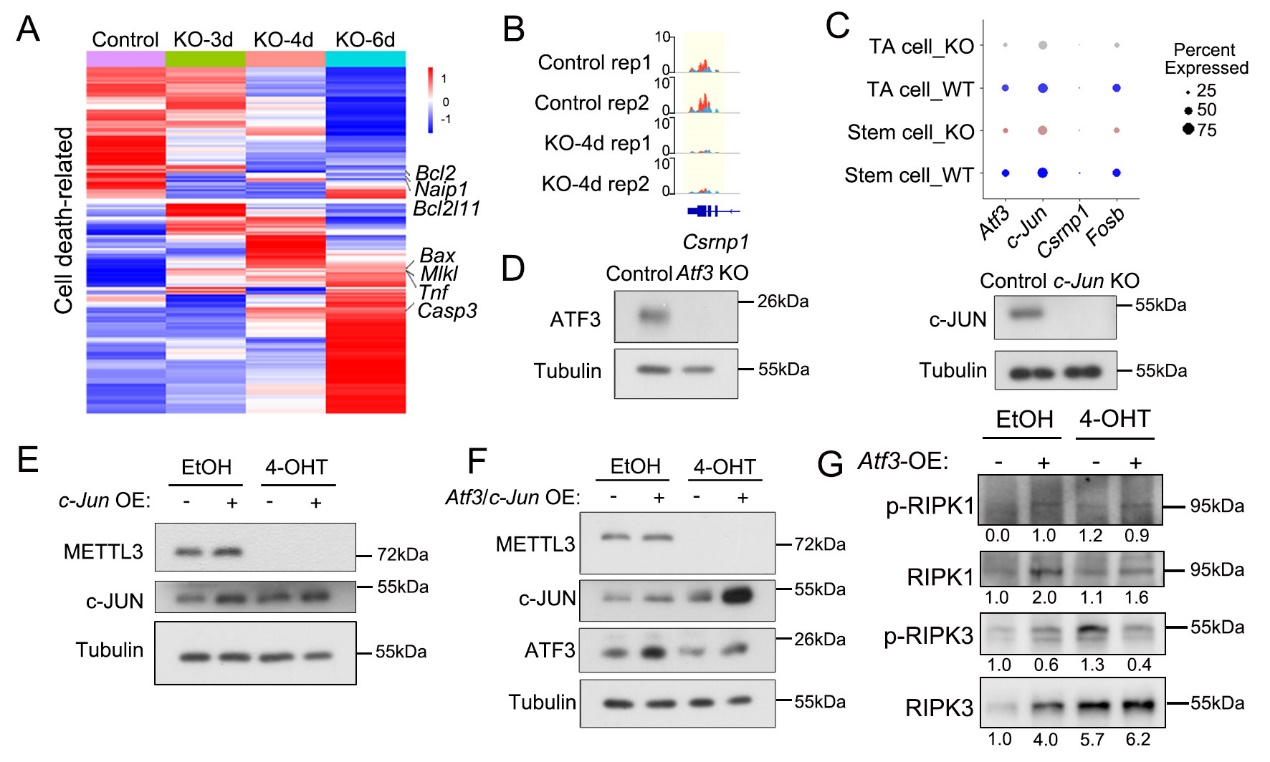


**Fig. S3 Identification of transcriptional factors that regulate cell death in the intestinal epithelium.** **(A)** Expression heatmap of altered cell death-associated genes in sorted ISCs from control and *Mettl3*-KO mice at the indicated time. **(B)** Integrative Genomics Viewer (IGV) tracks displaying MeRIP-seq reads along the TF *Csrnp1* in Lgr5^high^ ISCs of control and *Mettl3*-KO mice. Blue reads are from input libraries and red reads from anti-m^6^A immunoprecipitation libraries. The Y axis represents the CPM (count per million) of genes. The yellow boxes of the tracks depict the positions of m^6^A peaks. **(C)** scRNA-seq analysis showing the expression of *Atf3*, *c-Jun*, *Csrnp1*, and *Fosb* in stem cells and TA cells in control and *Mettl3*-KO mice. **(D)** Immunoblotting analysis of ATF3 and c-JUN protein in organoids derived from *Vil-CreERT2;Rosa26^loxp-stop-loxp-Cas9-EGFP^* mice, which were infected with recombinant AAV to knock out *Atf3, c-Jun*. Tubulin, loading control. Data were from one of three independent experiments. **(E, F)** Immunoblotting analysis of of ATF3 and c-JUN protein in organoids derived from *Vil-CreERT2*;*Mettl3^fl/fl^* mice and infected with *c-Jun-*expressing retrovirus (E) or *Atf3/c-Jun*-expressing retrovirus (F) at day 3 after EtOH or 4-OHT treatment for 2 days. Tubulin, loading control. **(G)** Immunoblotting and quantitative analyses of the activity of RIPK1 and RIPK3 in *Vil-CreERT2*;*Mettl3^fl/fl^* derived organoids. The organoids were treated with EtOH and 4-OHT for 2 days and infected with control or *Atf3*-expressed lentivirus. GAPDH, loading control. Data were from one of three independent experiments.

**Fig. S4**


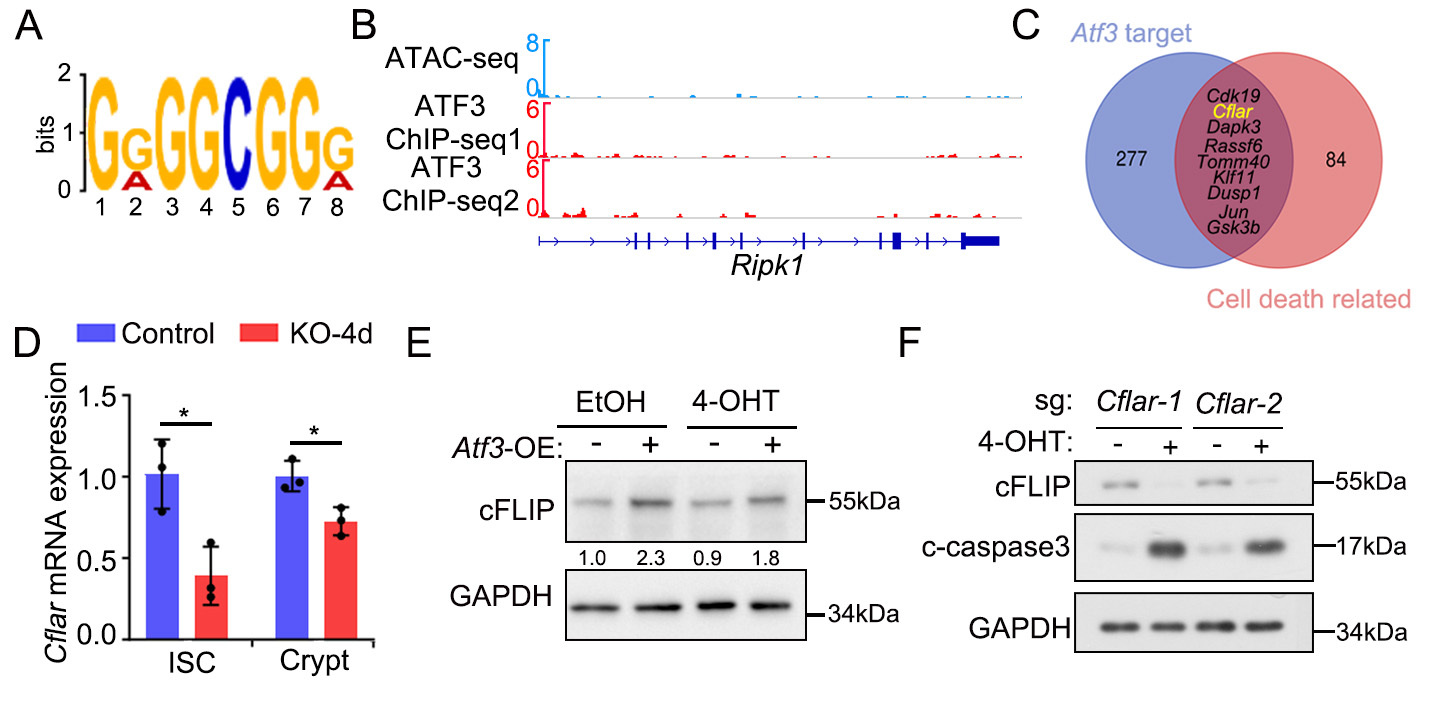


**Fig. S4 Identification of ATF3 target genes that regulate cell death in *Mettl3*-KO organoids.** **(A)** HOMER motif of ATF3 ChIP-seq. **(B)** ChIP-seq and ATAC-seq tracks of ATF3 binding to *Ripk1* gene loci. **(C)** Venn diagram depicting the overlap of *Atf3* target genes and differential expressed cell death-related genes in *Mettl3*-KO mice. **(D)** Expression of *Cflar* gene in FACS-sorted Lgr5^high^ ISCs and crypt cells from control and *Mettl3*-Lgr5-KO mice at 4 dpt by q-PCR. n=3 mice per group, each dot represents one mouse. **(E)** Immunoblotting and quantitative analyses of cFLIP in *Vil-CreERT2*;*Mettl3^fl/fl^* derived organoids. The organoids were treated with EtOH and 4-OHT for 2 days and infected with control or *Atf3*-expressed lentivirus. GAPDH, loading control. **(F)** Immunoblotting analysis of cFLIP protein in organoids derived from *Vil-CreERT2*;*Mettl3^fl/fl^* mice and infected with *Cflar-*expressing retrovirus at day 3 after EtOH or 4-OHT treatment for 2 days. GAPDH, loading control. Data were from one of three independent experiments.

**Fig. S5**


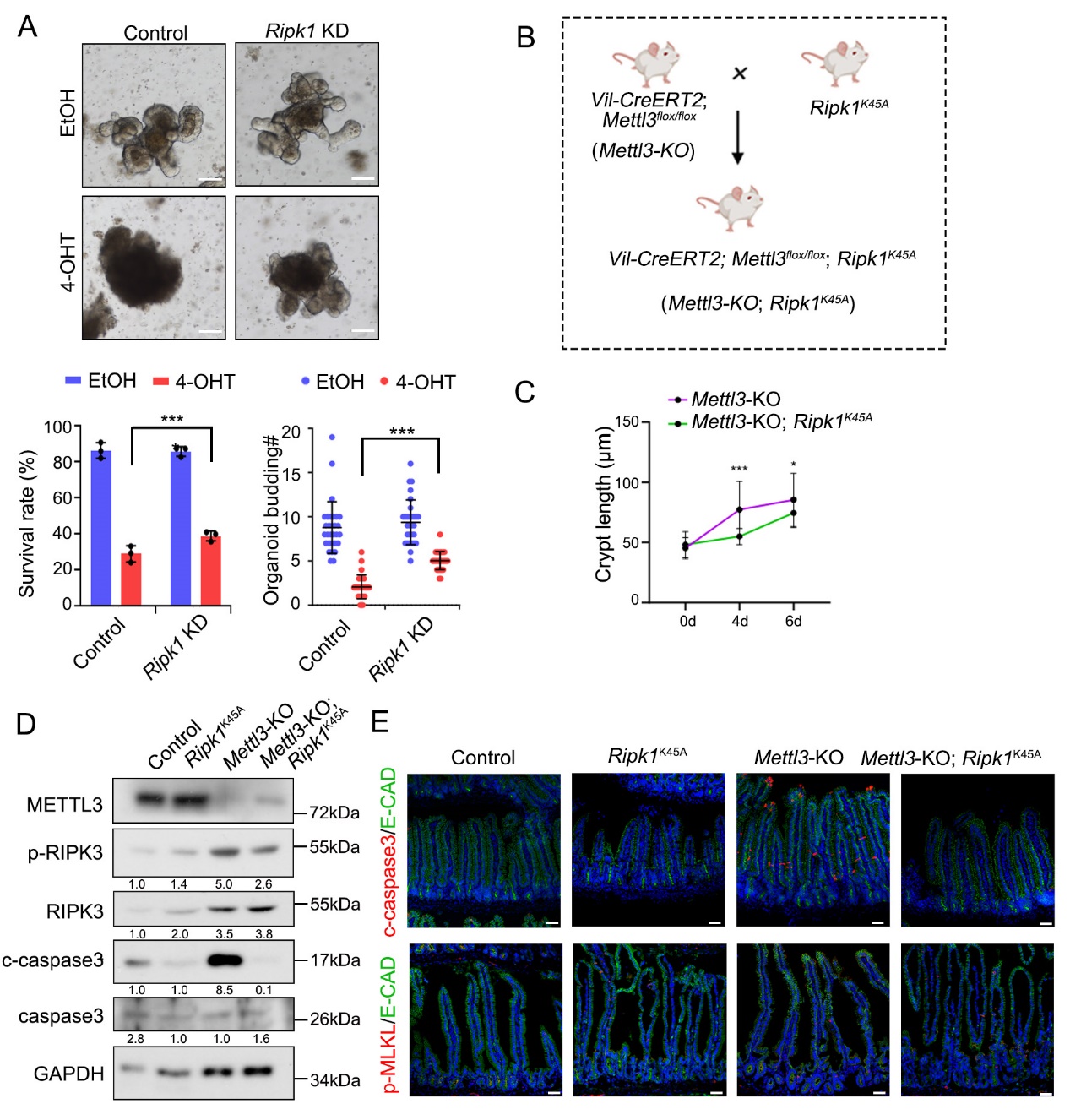


**Fig. S5 RIPK1 inactivation alleviates cell death in *Mettl3*-KO organoids and mice. (A)** Representative morphology (left), survival rate and budding number (right) of organoids derived from *Vil-CreERT2*;*Mettl3^fl/fl^* mice at 6 days after EtOH or 4-OHT treatment for 2 days and infected with lentivirus to knock down *Ripk1*. Data were from one of three independent experiments. **(B)** Schematic diagram of generating *Mettl3*-KO;*Ripk1^K45A^* (*Vil-CreERT2*;*Mettl3^fl/fl^*; *Ripk1^K45A^*) mice. **(C)** Quantitative analyses of crypt length in *Mettl3*-KO (*Vil-CreERT2*;*Mettl3^fl/fl^*) and *Mettl3*-KO;*Ripk1^K45A^* (*Vil-CreERT2*;*Mettl3^fl/fl^*;*Ripk1^K45A^*) mice at indicated times after injected with 20 mg/mL TAM. (n=3 mice/group). **(D)** Immunoblotting and quantitative analyses of cell death-associated protein expression in crypt epithelium of control, *Ripk1^K45A^*, *Mettl3*-KO and *Mettl3*-KO;*Ripk1^K45A^* mice at 4 dpt. GAPDH, loading control. Data were from one of three independent experiments. **(E)** Representative images of c-caspase 3^+^ cells and p-MLKL^+^ cells in jejunum epithelium of control, *Ripk1^K45A^*, *Mettl3*-KO and *Mettl3*-KO;*Ripk1^K45A^* mice at 4 dpt. n=3 mice/group. All the data represent mean±SD. The data were analyzed by Two-way ANOVA (A). *P<0.05, **P<0.01 and ***P<0.001. Scale bars: 100 μm (A), 50 μm (E). Nuclei were counter-stained with DAPI.

Table S1: sgRNA sequences.

Table S2: qPCR primer sequences.
